# Corporate digital transformation and strategic investments of construction industry in China

**DOI:** 10.1016/j.heliyon.2023.e17879

**Published:** 2023-07-01

**Authors:** Zhuohui Zhu, Shuying Ning

**Affiliations:** a Central-South Architectural Design Institute Co., Ltd., Wuhan 430000, China; b School of Economics and Management, Hubei University of Technology, Wuhan 430000, China

**Keywords:** Digitization, Informatization, Textual analysis, Corporate strategic investments, Innovation

## Abstract

Along with industrial revolution and upgrading technologies, all industries are changing their tradition ways of operation and management. However, the expanding construction industry is way behind this technological transformation. Traditional ways of design and operation have become obstacles in its transforming process. Construction industry has played indispensable role in China's economic growth for past decades, but now it should advance with the times. Recently, industrial enterprises have begun to adopt new concepts like digital construction and parametric design. But there still lacks evaluation on effectiveness of digital reform on construction enterprises. Therefore, this paper takes advantage of 2007–2018 panel data of construction industry in China, applies textual analysis to measure digital reform, and empirically testifies the effect of digital reform on strategic investments of construction enterprises. Results show that digital reform significantly increases corporate strategic investments, including R&D inputs and innovation outputs. This paper also discloses vital mechanism behind, namely, management foresight. Besides, we show that effect is stronger in districts with healthier relationships between enterprises and local governments. Corporate competence and future potential are also strengthened consequently. This paper provides empirical evidence about effect of digital reform on construction industry, and points out a possible direction for further industrial reform.

## 1 Introduction

Nowadays, China is undergoing a systematic transformation and its speed of economic growth has been much slower than before. Since the total investments in fixed assets of whole society is decreasing, development of construction industry significantly decreases as well. Even though the construction industry is the pillar industry in national economy, it must reform and adapt to new structural changes of supply-side reform. Instead of continuing its traditional low-efficient way, the construction industry has to innovate, upgrade and seek for new developing mode to satisfy the high-quality requirements nationwide.

As pointed out by Ronald Coase, one of the winners of Nobel Prize, the nature of corporation is an organizational mechanism to allocate resources instead of the market. In that sense, the key to strengthen firm-level competitiveness and sustainable development ability lies in the innovation and high efficiency in resource allocation. However, traditional construction companies stick to old ways of construction and operation, which lack motivation to innovate their technologies and upgrade. These drawbacks hinder the companies to achieve higher efficiency [1] and better quality.

The new trend of global informatization in 21st centuries has penetrated through every industry and stimulated numerous technological innovations. Corporations in every country have done their best to adapt to this trend and accelerate innovation, promoting economic development of their nation. Application prospect of worldwide informatization is leading to a large-scale industrial transformation and cross-border cooperation among industries. Since technological innovation is the main driving force to promote sustainable development of economy and society [2], Chinese people also put forward a strong demand for informatization and innovation. This trend especially challenges construction industry who have been used to traditional ways of operation and construction, but it also provides much more potential for this industry. The endeavor to innovate and digital transformation can improve firm's ability of resource allocation, productive efficiency [3] and ultimately enhance its competitiveness.

**Fig. 1:**
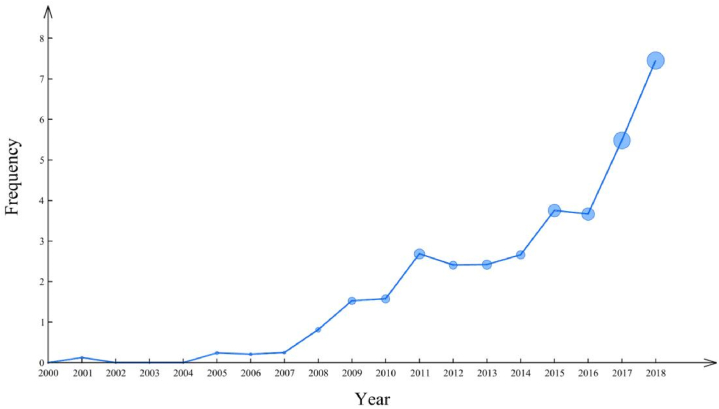
Year Trend of Phrases Frequency related to Digitalization.

Rapid growth of digital technology has brought huge challenges as well as big opportunities for traditional construction industry. Worldwide digital transformation leads to new demand in the market and points out new direction for industrial development and technological innovation [4]. As shown in Fig. 1, the frequency of phrases related to digitalization in corporate annual reports of construction industry is increasing steadily, which means that the influence of digital transformation on construction firms is rising year by year. Digital transformation is an irresistible general trend. Every round circle in the trendline represents the standard deviation of frequency in every year. Bigger radius of circle means bigger difference in frequency of phrases related to digitalization among firms in a specific year. As we can see from Fig. 1, with increasingly stronger influence of digitalization on construction industry, more firms have taken actions to adapt to the changing times.

Taking advantage of digital transformation, firms should gradually increase their efforts on R&D, create a sustainable innovative system and protect innovation achievements. Important technological innovations in construction industry such as BIM and fabricated building provide timely and accurate data for their construction and manufacture process, thus synchronizing information for all related parties and ensuring firm-level high efficiency, good quality as well as sustainability [5]. However, construction industry as a whole has been undergoing transformation of digitalization, and the effectiveness of independent R&D on design and construction process is still under exploration. It's meaningful and also necessary to show the positive influence of digital transformation on construction industry, which is exactly what we do in this paper, and the evidence can help stimulate firms in construction industry to endeavor to reform and adapt to digitalization.

Taking advantage of listed firms in construction industry from Chinese A stock market between 2007 and 2018, we empirically test the influence of digital reform on innovative investments of construction firms. Using innovation input as well as output to measure firm-level innovative investments, we find that digital reform increases corporate R&D investments, patents application and citation. Moreover, we furtherly examine core mechanism of manager foresight behind that effect. In cross-sectional analysis, evidence shows that promotion effect of digital reform on corporate innovative investments is stronger in districts where the relation is healthier between local governments and firms. In real-effect tests, we show that promotion of innovative investments brought by digital transformation indeed strengthens corporate ability of sustainable development and competitiveness.

We make potential contributions in the following three aspects: Firstly, we enrich the literature on the economic consequences of digital reform. Prior literatures have documented the influence of digitalization on corporate workflow, industrial upgrade and real economy [[6], [7], [8]]. Some researchers focused on the construction industry and found multiple technological revolutions and industrial upgrade based on digital reform [9,10]. However, little has been explored on the effect of digital reform on corporate long-term strategic investment of construction industries. Our paper has contributed to this literature by providing empirical evidence that digital reform can impose a positive effect on the innovative investments of construction companies.

Secondly, we contribute to the literature on the influencing factors of corporate innovation. Previous literature has extensively discussed from multiple aspects on the influencing factors of innovation, including legal institutions, social culture, capital market openness, investor characteristics and corporate features [[11], [12], [13]]. However, there is little research on the influence of digitalization. Taking advantage of textual analysis on firm-level reports, we provide a valuable measure to quantify the impact of digital reform on firms, which can help further exploration on influence of digital reform. Our paper hence testifies the positive influence of digital reform on corporate innovation and the mechanism behind, thus enriching the literature on corporate innovation.

Thirdly, analysis of our paper provides empirical support for construction firms to take actions and adapt to digital reform. The results and findings of the paper may be of some reference value to the policy-makers in charge of administration on construction industry.

## 2 Institutional background and hypothesis development

### 2.1 Institutional background

The construction industry has kept a constant and stable growth of high speed for last 40 years, and grown into a national pillar industry whose annual output value is more than 24 trillions. The market of construction industry is so large that it obtains more than 0.1 million firms and 50 million employees. However, with the slowing down of China's economic growth, people's enthusiasm on real estate is cooled down and thus construction firms can no longer gain as much revenue as before, some of which may even go bankrupted.

Most firms in construction industry stick to traditional ways of management [14] and have relatively low efficiency in resource allocation, which inevitably leads to limited enterprise benefits [15]. Innovation, upgrade and reform of Chinese construction industry are extremely urgent for improving its competitiveness and ability for sustainable development [16].

### 2.2 Literature review

Digital revolution and information technology have become powerful driving forces for real economy [3] and provided new impetus for many industries to innovate. In China, the industrial pattern has been changed thoroughly by digital infrastructure [7]. The revolution of digitalization has actively encouraged and accelerated industrial reform on the operating workflows to be more efficient [6], including decision-making, resource allocation, production, design and construction process [17]. The digital technology serves as an important instrument to boost social revolution and economic development.

As the pillar industry in China's real economy, the construction industry has encompassed multiple technological changes associated with digitalization and begun to subvert current working lifecycles [3,9]. This transformation encourages industrial adaption to digital technologies, eventual upgrade [17] and high-quality development. Tuhaise et al. [10] proposed that BIM model with real-time data can achieve cyber-physical integration, enabling real-time monitoring of assets and activities and improving decision-making. Zhang et al. [3] mentioned smart construction as an interconnected, intelligent and efficient construction model built on a high degree of digitalization. Other integrations like assembled buildings, digital technology system construction, and smart platform construction are also being developed nowadays. These relevant applications of digital technology have assisted construction enterprises to improve the quality, productivity and efficiency [10,18].

Previous literature has extensively discussed the influencing factors of innovation and its economic outcomes from macro-level to micro-level [11,12,19]. There are many influencing factors of innovation including legal institutions, social culture, capital market openness, investor characteristics and corporate features [13]. Moreover, the political science literature [20] suggests that local officials’ future promotion prospects depend on the economic performance in the region they govern. And innovation is the critical driving force of regional economic growth [21], thus becoming a critical aspect for government focus. Although R&D is expensive, risky and has long-return period, it frequently generates large positive spillovers to the whole entity [22], thus accelerating the development of companies [23] and the entire society.

### 2.3 Hypothesis development

Pursuit for high profits is one of driving forces for companies to upgrade. And technological innovation plays an indispensable role in promotion of corporate economic growth [23]. Investments in research and development can improve corporate innovation ability and core competitiveness in the market. Meanwhile, it can also accelerate corporate transformation and upgrade [17], thus providing sufficient power for future development. Therefore, if construction firms take actions to improve their technological innovations and transform labor-intensive industry into technology-intensive one, the digital reform will also help optimize corporate operational efficiency [18] and thus enhance corporate competitiveness.

In recent years, it's growing to be a common sense in construction industry to adapt to digital transformation [6]. This upgrade can help improve construction quality and program efficiency [24], alleviate the impact of risk and uncertainty, and thus enhance corporate synthetical strength [25]. Taking advantage of digital reform, construction industry can redefine its operation [3], reconstruct its ecosystem and transform fundamentally.

Most firms in construction industry independently research and try to develop technological innovation, thus renewing their process of manufacture as well as management through application of new technologies [7]. During the process of digital reform, construction industry can creatively explore new business model, and expand from traditional physical space to digital space, leading to a totally new industrial ecosphere. Technological innovation brought by digital reform can lead to high efficiency, accuracy and consistency of program operation management [9]. According to the analysis above, we make a theoretical hypothesis as following.

H1 Digital reform leads to improvement of innovative investments in construction firms.

Managers hold great power, influence corporate daily operation and make decisions on long-term strategies. In that sense, the transform from traditional mode to digital way needs huge investment [13,26] and inevitably solid support from managers [27]. Only after managers realize the value and advantage of digital transformation will they make strategic planning on innovation, stick to R&D process and improve corporate innovation ability [28]. It's of great importance for companies to accomplish digital upgrade of the entire process on design, construction and program maintenance, and thus reduce repetitive workload. With the manager foresight on digitalization, firms are able to improve their resource allocation efficiency, reduce operation uncertainty and enhance their competitiveness. According to the theoretical analysis, we make a hypothesis as following.

H2 Corporate manager foresight on digitalization leads to stronger positive effect of digital reform on corporate innovative investments.

## 3 Empirical analysis

### 3.1 Sample description and data source

We select listed firms of construction industry in Chinese A stock market between 2007 and 2018 as our main example. Furtherly, we drop ST and PT firms, and samples with negative owners’ equities. Variables on firm-level R&D, patents and controls are mainly from China Stock Market and Accounting Research Database (CSMAR), which is widely used by papers focusing on China background [29,30].

The data and measurement on digital reform are based on the textual analysis method. Firstly, we manually collect corporate annual financial reports of the listed firms over the entire sample period from the official website. Secondly, we use Python and its *pdfminer3k* library to transform the pdf file of annual reports into txt file. Thirdly, we imported the algorithm concerning string matching and statistics to do the word segmentation procedures and count the number of occurrences of digital-related phrases. And then, we take the natural logarithm of this phrase frequency to measure corporate digital reform.

### 3.2 Research design and variable definition

Following Bertrand and Mullainathan [31], Azoulay et al. [32] and Dimmock et al. [33], we construct generalized difference-in-differences model to explore the influence of digital reform on innovative investments of construction firms.

(1) $${Rd}_{it}/{Patent}_{it}/{Citation}_{it}=\beta_{0}+\beta_{1}{Revol}_{it}+{Controls}_{it}+Firm\;FE+Year\;FE+Province\;FE+\varepsilon_{it}$$

There are three main dependent variables in the model. The first one is *RD*_*it*_, which refers to firm-level investments on research and development of firm *i* in year *t* [23]. The second one is *Patent*_*it*_, which denotes the number of invention patents being applied and later authorized of firm *i* in year *t* [34]. The third variable is *Citation*_*it*_, which means the citation times of invention patents being applied and later authorized of firm *i* in year *t* [35]. The first variable, *RD*_*it*_ measures firm-level input on strategic research and development, and the latter two variables, *Patent*_*it*_ and *Citation*_*it*_, measure output on firm-level technological innovation. Therefore, our paper evaluates the influence of digital reform on corporate innovative investments from prospects of both R&D input and innovation output [36].

The main independent variable, *Revol*_*it*_, measures firm-level influence of digital reform shocks of firm *i* in year *t*. We manually collect and then textual analyze the annual reports of every listed firm in every year to get the frequency of digital-related phrases, which is exactly how we measure the effect of digital reform on specific firm. In order to identify the impact of digital reform on corporate strategical investments, we set treatment group as those firms who are under impact of digital reform, and control group is those who are under little or no impact of digital reform. Namely, the variable *Revol*_*it*_ takes the value of 0 before a firm is influenced by digital reform. After the firm is under impact of digital reform, *Revol*_*it*_ takes the value of influence degrees from digital reform. Besides, following the literature [37,38], *Controls*_*it*_ contains an extensive array of factors identified to be potential determinants of corporate innovative inputs and outputs. *Firm FE*, *Year FE* and *Province FE* represent fixed effects on firm-level, year-level and province-level respectively, aiming to control the influence of heterogeneities from these levels. *ε* represents the random disturbance item. We apply generalized DiD model with multi-level fixed effects to do the empirical analysis, and the coefficient of *Revol*_*it*_*, β*_*1*_*,* represents the influence of digital reform on corporate innovative investments. In order to alleviate the concern from outliers, we winsorize every variable at 1% and 99% level. Definition of main variables is demonstrated in Table 1.

**Table 1:** Variable definition.

Variable	Definition
*Rd*	Corporate investments on research and development, divided by prime operating revenues
*Patent*	Natural logarithm of the total number of invention patents filed by specific company and authorized eventually
*Citation*	Natural logarithm of total citation number made to the invention patents filed by specific firm and authorized eventually
*Revol*	Extent of influence of digital reform on specific firm, measured by the natural logarithm of corporate digital-related phrase frequencies
*Asset*	Natural logarithm of the scale on corporate fixed assets
*Roa*	Corporate return on assets
*Lev*	Corporate leverage, measured by liabilities divided by assets
*Rev*	Natural logarithm of the scale on corporate prime operating revenues
*Mc*	Corporate management expenses divided by prime operating revenues
*Fc*	Corporate financial expenses divided by prime operating revenues
*Noc*	Net cash flows from corporate operating activities divided by prime operating revenues
*Dual*	Dummy for firm-level duality of chairman and CEO
*Board*	Natural logarithm of board size
*IndBoard*	Ratio of independent boards
*Shareh1*	Shareholding ratio of corporate biggest shareholders
*Sharecon*	Dummy for connection among corporate biggest ten shareholders
*Stos*	Dummy for whether corporate ownership structure changes
*Emp*	Natural logarithm of the total number of corporate employees
*Age*	Firm age since listed
*Soe*	Dummy for corporate ownership property, state-owned or non-state-owned
Firm FE	Firm-level fixed effects
Year FE	Year-level fixed effects
Province FE	Province-level fixed effects

Table 2 demonstrates the descriptive statistics of our main variables. The mean value and standard deviation of *Rd* are 1.459 and 1.495, while its minimum and maximum are 0 and 4.481, respectively. The distribution of *Rd* means there is much variation among different samples, which is good to the following empirical analysis. The deviation among samples of *Patent* and *Citation* are also good and suitable for our study. The key independent variable, *Revol*, has a mean value of 1.001, standard deviation of 0.902, minimum of 0, and maximum of 3.219, which is also beneficial for our analysis. Besides, distribution of control variables is similar to the literature on corporate governance, which means that our sample is representative of all listed firms.

**Table 2:** Descriptive statistics of main variables.

Variable	Obs	Mean	Sd	Min	P25	P50	P75	Max
*Rd*	609	1.459	1.495	0.000	0.000	0.896	3.015	4.481
*Patent*	609	1.327	1.612	0.000	0.000	0.693	2.197	5.991
*Citation*	609	0.512	0.628	0.000	0.000	0.134	0.944	2.197
*Revol*	609	1.001	0.902	0.000	0.000	0.693	1.609	3.219
*Asset*	609	22.978	1.613	19.523	21.990	22.694	23.586	27.435
*Roa*	609	0.034	0.042	−0.136	0.015	0.027	0.049	0.182
*Lev*	609	0.666	0.151	0.183	0.573	0.687	0.787	0.894
*Rev*	609	22.393	1.814	17.908	21.363	22.093	23.037	27.247
*Mc*	609	5.504	4.150	1.420	2.920	4.500	6.670	28.800
*Fc*	609	1.693	2.197	−3.750	0.430	1.390	2.510	13.000
*Noc*	609	0.001	0.161	−0.589	−0.055	0.019	0.064	0.522
*Dual*	609	0.171	0.377	0.000	0.000	0.000	0.000	1.000
*Board*	609	2.249	0.150	1.792	2.079	2.303	2.303	2.639
*IndBoard*	609	0.394	0.078	0.333	0.333	0.364	0.429	0.667
*Shareh1*	609	0.384	0.154	0.045	0.272	0.368	0.497	0.726
*Sharecon*	609	0.535	0.499	0.000	0.000	1.000	1.000	1.000
*Stos*	609	0.662	0.474	0.000	0.000	1.000	1.000	1.000
*Emp*	609	7.941	1.719	4.220	6.738	7.789	8.632	12.555
*Age*	609	2.031	0.698	0.693	1.609	2.079	2.565	3.178
*Soe*	609	0.488	0.500	0.000	0.000	0.000	1.000	1.000

### 3.3 Main regression analysis: digital reform and corporate innovation

Table 3 shows the influence of digital reform on corporate R&D investment, patent application and citation. Dependent variables of Columns 1, 2, and 3 are *Rd*, *Patent* and *Citation*, respectively. The coefficients of digital reform variable (*Revol*) are significantly positive at 1% level in all three columns, indicating that digital reform leads to the increase in corporate innovative investments of construction firms. And the increase is reflected in corporate innovation inputs as well as outputs, thus verifying our main hypothesis H1. In a word, under the trend of digital revolution, construction companies will invest in corporate technologies and innovations, and thus strengthen the potential of corporate future growth and regional economic development.

**Table 3:** Digital reform and corporate innovative investments.

	(1)	(2)	(3)
	*Rd*	*Patent*	*Citation*
*Revol*	0.209***	0.269***	0.120***
	(4.214)	(2.874)	(4.574)
*Asset*	0.540**	0.660	0.317***
	(2.378)	(1.502)	(3.266)
*Roa*	0.139**	−1.583	1.566
	(2.274)	(−0.869)	(1.534)
*Lev*	−0.168	−0.373	0.384
	(−1.365)	(−0.591)	(1.173)
*Rev*	−0.660***	0.085	−0.196
	(−4.042)	(0.228)	(−1.320)
*Mc*	0.040	0.036	−0.000
	(1.332)	(0.444)	(−0.002)
*Fc*	0.040	0.029	0.003
	(0.738)	(1.031)	(0.154)
*Noc*	0.020	−0.019	−0.207
	(0.072)	(−0.449)	(−1.564)
*Dual*	−0.144	0.156	−0.021
	(−1.298)	(1.153)	(−0.184)
*Board*	−0.074	0.241	−0.195
	(−0.853)	(0.429)	(−0.603)
*IndBoard*	0.028	0.167*	−0.497
	(0.250)	(1.936)	(−0.728)
*Shareh1*	−0.185	1.092	0.782
	(−1.180)	(0.901)	(1.203)
*Sharecon*	−0.106	0.035	0.044
	(−0.835)	(0.585)	(0.377)
*Stos*	0.029	0.048	0.035
	(1.064)	(1.164)	(0.553)
*Emp*	−0.108	−0.107	−0.005
	(−0.704)	(−1.131)	(−0.098)
*Age*	0.073	0.489	0.007
	(0.263)	(1.526)	(0.030)
*Soe*	−0.327	−0.060	0.153
	(−0.239)	(−0.495)	(0.899)
*Constant*	15.289***	0.253	4.688
	(3.329)	(0.131)	(1.378)
*Firm_FE*	Yes	Yes	Yes
*Year_FE*	Yes	Yes	Yes
*Province_FE*	Yes	Yes	Yes
*Observations*	609	609	609
*R2*	0.80	0.87	0.57

Note: Coefficients of variables are in row 1, and the figures in brackets of the corresponding row 2 are the t-value of each variable. T-statistics reported in parentheses are robust to heteroskedasticity and clustered at the province level. ***, ** and * represent significance at 1%, 5% and 10% level, respectively. The following tables are the same.

### 3.4 Mechanism analysis: manager foresight channel

As described above, digital reform positively affects innovative investments of construction firms. And in this part, we will furtherly explore possible mechanisms behind this effect. As an important part of corporate governance, manager's decisions will impose great and long-term influence on business development. Therefore, if managers realize the significance of digital reform and emphasize its huge potential, they will actively adjust corporate development strategies and put more efforts into technologies and innovation, in order to achieve digital transformation and industrial upgrade.

We manually collect all reports on MD&A, namely, management discussion and analysis, and run similar textual analysis procedures on those reports. In this process, we identify the frequency of digital-related contents and thus define the *MaView* variable to measure manager's broad and long-term foresight on digital reform. Our regression results are shown in Table 4, in which dependent variables of Columns 1, 2, 3 and 4 are *MaView*, *Rd*, *Patent* and *Citation*, respectively. The coefficient of *Revol* is positive and significant at 1% level in Column 1, indicating that corporate managers attach great importance to digital-related concepts after the digital reform. And in Columns 2, 3 and 4, we include *Revol*, *MaView* and their interaction term, *Revol_MaView* to do further analysis. We find that coefficients of *Revol_MaView* are all significantly positive in Columns 2, 3 and 4, showing that when corporate managers attach more importance to digital-related concepts, the promotion effect of digital reform on corporate innovative investments will be stronger, thus proving hypothesis H2. With the attention shift of managers to technological upgrade, digital reform can have an intensified influence on corporate innovation, which enriches the research on digitalization and innovation from the perspective of corporate management.

**Table 4:** Digital reform, manager foresight and corporate innovative investments.

	(1)	(2)	(3)	(4)
	*MaView*	*Rd*	*Patent*	*Citation*
*Revol*	0.734***	0.086**	0.033	0.051**
	(8.371)	(2.756)	(0.614)	(2.223)
*Revol_MaView*		0.093***	0.144**	0.042***
		(4.081)	(2.637)	(5.087)
*MaView*		−0.020	0.024	0.051***
		(−0.715)	(0.539)	(3.195)
*Asset*	−0.045	0.548**	0.689	0.311***
	(−0.881)	(2.409)	(1.499)	(3.497)
*Roa*	1.366***	0.139**	−1.872	1.460
	(3.102)	(2.270)	(−0.763)	(1.425)
*Lev*	−0.661**	−0.168	−0.232	0.434
	(−2.833)	(−1.329)	(−0.361)	(1.344)
*Rev*	0.009	−0.655***	0.096	−0.203
	(0.080)	(−4.125)	(0.314)	(−1.387)
*Mc*	0.007	0.041	0.036	−0.002
	(1.057)	(1.374)	(0.669)	(−0.137)
*Fc*	−0.017	0.040	0.032	0.005
	(−1.182)	(0.746)	(1.180)	(0.259)
*Noc*	0.006	0.032	−0.016	−0.225
	(0.053)	(0.117)	(−0.416)	(−1.676)
*Dual*	0.028	−0.141	0.164	−0.035
	(0.352)	(−1.279)	(1.702)	(−0.315)
*Board*	0.443	−0.082	0.055	−0.150
	(1.159)	(−0.948)	(0.105)	(−0.479)
*IndBoard*	−0.021	0.027	0.170*	−0.453
	(−0.382)	(0.246)	(1.849)	(−0.725)
*Shareh1*	−0.342	−0.189	1.114*	0.851
	(−0.671)	(−1.180)	(1.732)	(1.306)
*Sharecon*	−0.026	−0.108	0.039	0.050
	(−0.979)	(−0.849)	(1.069)	(0.433)
*Stos*	−0.086**	0.030	0.058	0.039
	(−2.715)	(1.072)	(1.260)	(0.648)
*Emp*	0.033	−0.101	−0.102	−0.018
	(0.702)	(−0.678)	(−1.599)	(−0.359)
*Age*	0.056	0.053	0.444	0.031
	(0.816)	(0.188)	(1.284)	(0.141)
*Soe*	−0.041	−0.318	−0.045	0.147
	(−0.748)	(−0.234)	(−0.425)	(0.884)
*Constant*	0.118	15.239***	0.700	4.814
	(0.100)	(3.428)	(0.390)	(1.446)
*Firm_FE*	Yes	Yes	Yes	Yes
*Year_FE*	Yes	Yes	Yes	Yes
*Province_FE*	Yes	Yes	Yes	Yes
*Observations*	609	609	609	609
*R2*	0.84	0.80	0.87	0.57

### 3.5 Heterogeneity analysis: influence from relation between governments and business

In China, relation between local governments and companies is of vital importance to corporate survival and development. Whether the relation is healthy decides corporate strategic tendency of short-term investing or long-term planning. In recent years, relation between local governments and business has been a hot topic in conferences from all levels of governments, and many policies have been introduced to shape increasingly closer and more cleaning relation between governments and corporations within districts. Therefore, we divide the sample into two subgroups by the mean value of health degree in each city and examine heterogeneity in the influence of digital reform on corporate innovative investments.

The results are demonstrated in Table 5. Columns 1, 3 and 5 show the results of subgroup with much healthier relation, while Columns 2, 4 and 6 show the results of subsample with less healthier relation. We can see from Table 5 that the coefficient of *Revol* is significantly positive in subsample with much healthier relation, while not significant in subgroup of less healthier relation. In a word, improvement of relation towards a closer and more cleaning direction between governments and corporations can promote corporate tendency into more long-term developing strategies and innovation investment plans under the tide of digital reform.

**Table 5:** Digital reform, relation between governments and business, and corporate innovative investments.

	(1)	(2)	(3)	(4)	(5)	(6)
	*Rd*	*Rd*	*Patent*	*Patent*	*Citation*	*Citation*
	*More*	*Less*	*More*	*Less*	*More*	*Less*
*Revol*	0.127***	0.086	0.213***	0.032	0.166***	0.022
	(3.133)	(1.110)	(3.201)	(0.340)	(3.394)	(0.323)
*Asset*	0.482*	0.304	0.169	0.244	0.562***	−0.050
	(1.956)	(1.255)	(0.600)	(0.767)	(3.011)	(−0.264)
*Roa*	0.156***	−0.373	−0.119	−1.529	0.068	2.067*
	(6.218)	(−0.158)	(−0.699)	(−0.820)	(0.113)	(1.828)
*Lev*	−0.145	−0.583	−1.158***	0.597	0.016	0.044
	(−1.548)	(−0.365)	(−8.626)	(0.767)	(0.245)	(0.087)
*Rev*	−0.838***	−0.576*	0.686***	−0.062	−0.312	−0.213
	(−5.760)	(−1.798)	(7.926)	(−0.236)	(−1.587)	(−0.783)
*Mc*	0.034*	0.024	0.113**	−0.005	−0.078*	0.014
	(2.015)	(0.463)	(2.398)	(−0.169)	(−2.057)	(0.626)
*Fc*	0.037	−0.037	0.035	0.041	−0.007	−0.001
	(0.541)	(−0.764)	(1.494)	(0.728)	(−1.025)	(−0.009)
*Noc*	−0.075	−0.366	0.012	−0.240	−0.040***	−0.088
	(−0.329)	(−0.939)	(0.255)	(−1.081)	(−3.342)	(−0.369)
*Dual*	0.088	−0.680**	0.099	0.058	0.031	−0.152
	(1.493)	(−2.371)	(0.795)	(0.381)	(0.510)	(−0.885)
*Board*	−0.073	−1.143	−0.296	0.195	0.017	−0.784**
	(−0.935)	(−0.545)	(−0.368)	(0.320)	(0.049)	(−2.759)
*IndBoard*	0.009	−2.036	0.156	0.486	0.036	−1.684
	(0.133)	(−0.624)	(1.528)	(0.207)	(1.060)	(−1.264)
*Shareh1*	−0.441**	0.635	1.478***	1.071	−0.015	0.397
	(−2.593)	(0.384)	(3.734)	(1.575)	(−0.183)	(0.383)
*Sharecon*	−0.239***	0.124	0.053	0.055	0.092***	−0.085
	(−6.525)	(0.841)	(1.275)	(0.543)	(3.274)	(−0.659)
*Stos*	0.044	−0.062	0.049	0.078	−0.043	0.046
	(0.789)	(−0.553)	(0.479)	(0.530)	(−0.671)	(0.358)
*Emp*	−0.231	−0.106	−0.012	−0.230*	−0.023	0.046
	(−0.907)	(−0.541)	(−0.127)	(−1.826)	(−0.230)	(0.268)
*Age*	0.327*	−0.289	0.475	0.448	0.339	−0.077
	(1.993)	(−0.544)	(1.553)	(1.470)	(0.715)	(−0.412)
*Soe*	0.812	−3.044***	0.012	−0.071	0.351	0.220
	(0.621)	(−3.958)	(0.150)	(−0.139)	(1.363)	(0.589)
*Constant*	8.608	12.246*	−2.184	−3.935	7.146*	8.170***
	(1.513)	(1.975)	(−0.518)	(−0.691)	(1.859)	(3.069)
*Firm_FE*	Yes	Yes	Yes	Yes	Yes	Yes
*Year_FE*	Yes	Yes	Yes	Yes	Yes	Yes
*Province_FE*	Yes	Yes	Yes	Yes	Yes	Yes
*Observations*	293	316	293	316	293	316
*R2*	0.84	0.83	0.92	0.76	0.70	0.52

### 3.6 Real effect tests: digital reform and corporate long-term development

It remains a question whether improvement of innovation brought by digital reform truly leads to the enhancement of corporate value and future growth potential. In this part, we choose three aspects to testify real effect of digital reform. The first variable we define is *Intg*, which equals the difference of total intangible assets in current period and prior period, divided by total assets. The second variable we construct is *Tobinq*, which equals the ratio of corporate market value and total assets. And the third variable is *Susgrow*, equaling return on net assets plus earnings retention ratio, divided by the difference of 1 and return on net assets plus earnings retention ratio. The regression results are shown in Table 6, and the coefficients of *Revol* are significantly positive in these three columns. Namely, the improvement of innovative investments brought by digital reform truly leads to the enhancement of corporate value and sustainable growth ability.

**Table 6:** Digital reform and corporate long-term development.

	(1)	(2)	(3)
	*Intg*	*Tobinq*	*Susgrow*
*Revol*	0.217***	0.128**	0.009***
	(3.026)	(2.299)	(3.588)
*Asset*	0.942	−0.596***	0.007
	(1.288)	(−3.935)	(0.608)
*Roa*	−1.037	0.107	0.076***
	(−0.839)	(1.427)	(9.825)
*Lev*	−0.288	0.216	0.117***
	(−1.086)	(0.983)	(3.366)
*Rev*	0.180	0.492**	0.003
	(0.216)	(2.138)	(0.319)
*Mc*	−0.084	0.050**	−0.001
	(−0.393)	(2.576)	(−0.433)
*Fc*	−0.029	0.038	0.001
	(−0.405)	(0.918)	(0.392)
*Noc*	−0.960	0.539*	0.000
	(−1.084)	(1.937)	(0.011)
*Dual*	0.016	0.212*	−0.002
	(0.139)	(2.036)	(−0.327)
*Board*	−1.051	0.371	−0.040**
	(−0.650)	(1.529)	(−2.129)
*IndBoard*	−2.733	0.016	−0.001
	(−0.698)	(0.337)	(−0.031)
*Shareh1*	3.763	−0.124	0.024
	(1.093)	(−0.863)	(0.549)
*Sharecon*	0.026	−0.049	−0.006
	(0.114)	(−0.405)	(−0.972)
*Stos*	0.230**	0.033	−0.003
	(2.216)	(1.120)	(−1.056)
*Emp*	−0.142	0.096	−0.001
	(−0.361)	(1.328)	(−0.032)
*Age*	0.553	0.088	0.008
	(0.615)	(0.987)	(0.758)
*Soe*	2.778	−0.262	0.026
	(0.944)	(−0.603)	(0.922)
*Constant*	−22.037	14.076***	−0.167
	(−1.046)	(4.221)	(−0.758)
*Firm_FE*	Yes	Yes	Yes
*Year_FE*	Yes	Yes	Yes
*Province_FE*	Yes	Yes	Yes
*Observations*	609	609	609
*R2*	0.36	0.85	0.86

### 3.7 Parallel trend analysis

The precondition of difference-in-differences regression lies in the parallel trend assumption. Namely, the variation trend of treatment group and control group should be similar before the shock happens. According to Moser and Voena [39], we construct a variable, *Parallel*, to test the assumption. For treatment group, *Parallel* equals to 0 in the second year before the shock, and 1 in the first year before the shock. And for the control group, it equals to 0 in the first and second year before the shock. The regression results are demonstrated in Table 7, and coefficients of *Parallel* are not significant in all three columns. In a word, there is no evidence to show difference in variation trend of treatment group and control group before the shock. Therefore, parallel trend assumption is satisfied and it provides more proof for our analysis.

**Table 7:** Parallel trend analysis.

	(1)	(2)	(3)
	*Rd*	*Patent*	*Citation*
*Parallel*	−0.399	−0.194	−0.099
	(−1.363)	(−0.586)	(−0.445)
*Asset*	−0.458	−1.264	−0.661
	(−0.409)	(−1.180)	(−0.619)
*Roa*	−4.175	9.427	7.218
	(−0.384)	(0.753)	(0.683)
*Lev*	−1.145	−1.077	−2.117
	(−0.284)	(−0.341)	(−0.654)
*Rev*	0.959	1.995	1.021
	(1.013)	(1.475)	(1.403)
*Mc*	−0.028	0.059	0.048
	(−0.290)	(0.650)	(0.756)
*Fc*	0.007	−0.018	0.058
	(0.042)	(−0.067)	(0.217)
*Noc*	−0.375	−0.062	0.017
	(−0.281)	(−0.079)	(0.021)
*Dual*	1.159	0.721	1.131
	(1.485)	(0.729)	(1.227)
*Board*	3.158	0.953	−0.036
	(1.503)	(0.400)	(−0.025)
*IndBoard*	7.293**	−5.130	−3.952
	(2.235)	(−0.965)	(−1.056)
*Shareh1*	0.236	6.061*	−0.328
	(0.072)	(1.787)	(−0.182)
*Sharecon*	0.252	0.536	0.418
	(0.540)	(0.970)	(0.477)
*Stos*	−0.091	0.309	0.359
	(−0.261)	(0.651)	(0.773)
*Emp*	−1.196	−1.324*	−0.198
	(−1.124)	(−1.777)	(−0.238)
*Age*	0.720	1.175	−0.055
	(0.635)	(0.801)	(−0.045)
*Soe*	0.127	−2.677	−1.190
	(0.061)	(−1.096)	(−0.656)
*Constant*	−14.151	−9.143	−2.698
	(−0.791)	(−0.601)	(−0.181)
*Firm_FE*	Yes	Yes	Yes
*Year_FE*	Yes	Yes	Yes
*Province_FE*	Yes	Yes	Yes
*Observations*	108	108	108
*R2*	0.97	0.97	0.86

## 4 Conclusion

This study takes in the listed firms of construction industry in Chinese A stock market between 2007 and 2018, and empirically test the influence of digital reform on innovative investments of construction firms. Using innovation inputs as well as outputs to measure firm-level innovative investments, we find that digital reform increases corporate innovative investments. Moreover, we examine a core mechanism of manager foresight behind that effect. In cross-sectional analysis, evidence shows that promotion effect of digital reform on firm-level innovative investments is stronger in districts where relation is healthier between local governments and firms within. In real-effect tests, we show that promotion of innovative investments brought by digital transformation indeed strengthens corporate ability of sustainable development and competitiveness.

Technological upgrade and industrial transformation brought by digital reform will gradually influence numerous fields in engineering construction and promote technological revolution in construction industry. Corporate managers should attach great importance to digital reform and thus promote innovation revolution hereafter, improving corporate future potential and competitiveness. This study not only enriches literature in related fields theoretically, but also empirically testifies the positive effect of corporate actions in adapting to digital reform. We provide some quantified evidence for construction industry in the tide of digital reform, and also offer much reference for governmental administrative department that certain policies should be introduced to stimulate corporate digital transformation and innovation.

Due to the limitation of big data and empirical analysis, we are not able to explore the multiple stages of change happened within a specific company facing the digital reform and its corresponding strategic adjustments or internal control adaption. In future research, several case studies are highly recommended to do the relevant exploration.

## Author contribution statement

Zhuohui Zhu: Performed the experiments; Analyzed and interpreted the data; Wrote the paper.

Shuying Ning: Conceived and designed the experiments; Analyzed and interpreted the data; Contributed reagents, materials, analysis tools or data; Wrote the paper.

## Data availability statement

Data will be made available on request.

## Declaration of competing interest

The authors declare that they have no known competing financial interests or personal relationships that could have appeared to influence the work reported in this paper.
